# Refining the clinical utility of [^177^Lu]Lu/[^225^Ac]Ac-PSMA tandem RLT in patients with metastatic castration resistant prostate cancer

**DOI:** 10.1007/s00259-025-07632-1

**Published:** 2025-11-01

**Authors:** Liam Widjaja, Johannes Hornfeck, Sophie C. Siegmund, Franz J. Gildehaus, Nina-Sophie Schmidt-Hegemann, Vera Wenter, Gabriel T. Sheikh, Konrad Klimek, Jozefina Casuscelli, Christian G. Stief, Mathias J. Zacherl, Rudolf A. Werner

**Affiliations:** 1 Department of Nuclear Medicine, LMU University Hospital, LMU Munich, Marchioninistr. 15, Munich, D-81377 Germany; 2 Bavarian Cancer Research Center (BZKF), partner site Munich, Munich, Germany; 3 Department of Radiation Oncology, LMU University Hospital, LMU Munich, Munich, Germany; 4 Department of Urology, LMU University Hospital, LMU Munich, Munich, Germany; 5 The Russell H Morgan Department of Radiology and Radiological Sciences, Division of Nuclear Medicine, Johns Hopkins School of Medicine, Baltimore, USA; 6 German Cancer Consortium (DKTK), partner site Munich, a partnership between DKFZ and LMU University Hospital Munich, Munich, Germany

**Keywords:** PSMA-RLT, Tandem RLT, Prostate cancer, Targeted alpha therapy

## Abstract

**Purpose:**

This study aimed to evaluate the efficacy and safety of Lutetium-177/Actinium-225 prostate-specific membrane antigen tandem radioligand therapy ([^177^Lu]Lu/[^225^Ac]Ac-PSMA tandem RLT) and to explore clinical and imaging-based predictors of treatment response to support individualized patient selection.

**Methods:**

This retrospective, single-center study included 23 patients with mCRPC who underwent Fluor-18 ([^18^F]F)-PSMA-1007 positron emission tomography/computed tomography (PET/CT) and subsequent tandem RLT. Whole-body tumor segmentation on PET/CT and standard laboratory values were acquired before treatment initiation. Primary endpoint was partial response (PR), defined as either a decline in prostate specific antigen of ≥ 50% (according to prostate cancer clinical trial working group) or PET-based response according to Response Evaluation Criteria on PSMA PET/CT. Safety assessment included renal and hematological side effects following common terminology criteria of adverse events version 5.

**Results:**

Following two cycles of tandem RLT, 11 patients (48%) achieved a PR. The treatment was generally tolerated well. Grade 3 events included renal impairment in two (9%) and grade 3 anemia in five (22%) patients, while no Grade 4/5 events occurred. Patients with increased PSMA expression on pretherapeutic PET (defined by the average mean standardized uptake value of all tumor lesions [SUV_mean_]) had a higher response rate (86%; 6 out of 7) compared to those with decreased SUV_mean_ (31%; 5 out of 16). In Cox regression analysis, SUV_mean_ was significantly associated with PR with a hazard ratio of 1.34 (95% CI, 1.01–1.77; *P* = 0.042). PSMA-tumor volume (*P* = 0.036) and total lesion-PSMA (*P* = 0.041) were also significant predictors, whereas none of the clinical parameters showed predictive value. Kaplan-Meier analysis further confirmed SUV_mean_ as the strongest PR (*P* = 0.003).

**Conclusion:**

[^177^Lu]Lu/[^225^Ac]Ac-PSMA tandem RLT may offer a safe, effective treatment option. Assessment of PSMA expression on pretherapeutic PET predicts response, supporting its use in guiding personalized treatment.

## Introduction

Metastatic castration resistant prostate cancer (mCPRC) remains a major clinical challenge [1]. Especially in advanced stages of disease progression treatment options are limited [1]. In recent years, prostate-specific membrane antigen (PSMA)-targeted radioligand therapy (RLT) has emerged as a promising treatment option, particularly using the β^−^-emitter Lutetium-177 [2–4]. Unfortunately, treatment response under Lutetium-177 [^177^Lu]Lu-PSMA RLT is heterogenous with about 46% reaching partial response (defined as PSA-decline ≥ 50%) and overall survival limited to 15.3 months even under [^177^Lu]Lu-PSMA RLT [2].

Facing these challenges, targeted alpha therapy using the α-emitter Actinium-225, has been proposed, enabling enhanced cytotoxic potential due to its higher linear energy transfer (LET) and shorter tissue penetration range [5, 6]. Tandem approaches combining [^177^Lu]Lu-PSMA and Actinium-225 ([^225^Ac]Ac)-PSMA may offer safety and efficacy by exploiting both radionuclides [7]. Preliminary results of first clinical trials about [^177^Lu]Lu/[^225^Ac]Ac-PSMA tandem RLT demonstrated promising response rates even in patients previously progressing under monotherapy with [^177^Lu]Lu-PSMA RLT [7–9]. However, even under tandem RLT treatment responses vary among patients, underscoring the need for predictive indicators to guide the treating physician in patient selection to optimize therapeutic outcomes [8].

To date, evidence on predictors of response to tandem-RLT remains limited. In contrast, numerous studies have focused on identifying predictors of treatment response to monotherapy with [^177^Lu]Lu-PSMA RLT [10]. Among these, PSMA expression assessed on pretherapeutic PSMA-targeted positron emission tomography/computed tomography (PET/CT) emerged as the strongest and most consistent predictor for outcome [11, 12]. Nonetheless, utility in the setting of tandem RLT, particularly among patients who have previously progressed under [^177^Lu]Lu-PSMA RLT monotherapy, remains unknown.

Thus, the present study aims to identify clinical and imaging-based predictors of treatment response in patients with mCRPC scheduled for [^177^Lu]Lu/[^225^Ac]Ac-PSMA tandem RLT. As such, we compare the predictive value of PET/CT-derived parameters with clinical markers, subsequently seeking to establish predictors to support individualized treatment planning. Moreover, we aim to report on common adverse events under tandem RLT including chronic kidney disease (CKD) and hematological side effects.

## Materials and methods

### Patient population

In this retrospective, single-center study, 23 patients with a mean age of 74 ± 7 years suffering from mCRPC scheduled for [^177^Lu]Lu/[^225^Ac]Ac-PSMA tandem RLT were included (Table 1). All patients had progressive disease despite multiple previous treatments including radical prostatectomy, radiation therapy, androgen deprivation therapy and next-generation hormonal agents. Most had also received previous chemotherapy and [^177^Lu]Lu-PSMA RLT. Tandem RLT was carried out in accordance to the German Medicinal Products Act, AMG § 13.2b and the Declaration of Helsinki. This study was approved by the institutional review board (25–0130). Data from this patient cohort has been previously published, without analyzing predictors of treatment response [8].

**Table 1:** Patient characteristics (*n* = 23)

Variable (mean ± SD)	
Age	74 ± 7
Gleason score	8 ± 1
Previous treatments (%)	
Radical prostatectomy	63
Primary radiation therapy	17
Androgen deprivation therapy	100
Enzalutamide	91
Abiraterone acetate	90
Previous chemotherapy	95
Docetaxel	95
Cabazitaxel	75
[^177^Lu]Lu-PSMA RLT	74
Cycles (n=)	3 ± 3
Standard laboratory value	
Hb (g/dl)	10.6 ± 1.9
WBC (×10^3^/µL)	6.4 ± 2.8
Platelets (×10^3^/µL)	244 ± 80
eGFR (ml/min/1.73m^2^)	75 ± 20
AST (U/I)	45 ± 48
ALT (U/I)	21 ± 15
AP (U/I)	194 ± 207
LDH (U/I)	440 ± 485
PSA (µg/L)	298 ± 345
PSA doubling time (months)	3.4 ± 4.1
Site of tumor lesions (%)	
Osseous	96
Lymph nodes	83
Hepatic	22
Prostate bed	17
PET-derived parameters	
SUV_max_	18.01 ± 10.46
SUV_mean_	7.4 ± 2.05
PSMA-TV (ml)	799 ± 792
TL-PSMA (ml)	8666 ± 10,581

*Lu* Lutetium, *PSMA* prostate-specific membrane antigen, *RLT* radioligand therapy, *PFS* progression-free survival, *Hb* hemoglobin, *WBC* white blood cell count, *eGFR* estimated glomerular filtration rate, *AST* aspartate transaminase, *ALT* alanine transaminase, *AP* alkaline phosphatase *LDH* lactate dehydrogenase, *PSA* prostate-specific antigen, *PET* positron-emission tomography, *SUV* standardized uptake value, *TV* tumor volume, *TL* total lesion

### Assessment of PET-derived parameters

Prior to initiation of [^177^Lu]Lu/[^225^Ac]Ac-PSMA tandem RLT, all patients underwent [^18^F]F-PSMA-1007 PET/CT. Each patient received 228 ± 28 MBq of [^18^F]F-PSMA-1007. Imaging was performed one hour post-injection and included a helical CT (120 kV) followed by a PET acquisition from the vertex to mid-thigh (2.5 min per bed position). PET images were reconstructed using an iterative TrueX algorithm (three iterations, 21 subsets) with Gaussian post-reconstruction smoothing (2 mm full width at half-maximum), as previously described [13]. Image analysis was performed using Affinity 4.0.2 software (Hermes Medical Solutions, Stockholm, Sweden).

Whole-body tumor segmentation was performed according to previously established protocols [11, 14]. Briefly, lesions identified on pretherapeutic [^18^F]F-PSMA-1007 PET/CT were manually segmented using a standardized uptake value (SUV) threshold of 4. For each lesion, maximum SUV (SUV_max_), mean SUV (SUV_mean_), PSMA-tumor volume (PSMA-TV) and total lesion-PSMA (TL-PSMA, defined as SUV_mean_ x PSMA-TV) were determined. Finally, for patient-based analysis average SUV_max_, average SUV_mean,_ summed PSMA-TV and summed TL-PSMA were calculated [11].

### [^177^Lu]Lu/[^225^Ac]Ac-PSMA tandem RLT

[^177^Lu]Lu/[^225^Ac]Ac-PSMA tandem RLT was administered in accordance with a previously established treatment protocol [8]. Each treatment cycle involved the intravenous co-administration of 1982 ± 1434 MBq of [^177^Lu]Lu-PSMA and 5.95 ± 1.56 MBq of [^225^Ac]Ac-PSMA. Treatment cycles were repeated at intervals of 59 ± 21 days. Median number of treatment cycles was two (Interquartile range, 2–4) per patient.

### Assessment of clinical outcome

Patients undergoing [^177^Lu]Lu/[^225^Ac]Ac-PSMA tandem RLT were closely monitored throughout the treatment course. Laboratory evaluations including PSA levels, estimated glomerular filtration rate (eGFR) and blood cell counts were performed prior to and 4–6 weeks after each treatment cycle to assess treatment response and detect potential toxicities. In addition, [^18^F]F-PSMA-1007 PET/CT was routinely conducted after every two treatment cycles to evaluate imaging-based response.

Treatment response was assessed using both biochemical and imaging criteria. The Prostate Cancer clinical trial Working Group criteria version 3 (PCWG 3) [15] were applied for biochemical response evaluation, while imaging-based response was determined according to Response Evaluation Criteria in PSMA PET/CT (RECIP 1.0) [16].

The primary endpoint of the study was defined as a partial response (PR) following two cycles of tandem RLT, characterized by a PSA reduction of ≥ 50% and/or a PSMA-TV reduction of ≥ 30% in the absence of new lesions on PET/CT [16]. For patients who discontinued tandem RLT prior to the follow-up after two treatment cycles, the last available PSA value was used for response evaluation. Additionally, time to partial response was defined as the interval between the first RLT administration and the first documented evidence of partial response [15, 16].

Safety was assessed by monitoring adverse events, including chronic kidney disease (CKD), anemia, leukocytopenia and thrombocytopenia. These were graded according to Common Terminology Criteria for Adverse Events (CTCAE), version 5.0 [17]. To assess tolerability, we also evaluated clinical symptoms such as xerostomia, loss of appetite, nausea, fatigue and pain. CKD, anemia, leukocytopenia and thrombocytopenia were also assessed at last available follow-up, which was 55 days (median; IQR, 32–92 days) after the last cycle of [^177^Lu]Lu/[^225^Ac]Ac-PSMA tandem RLT.

### Statistical analysis

For statistical analysis we used GraphPadPrism 10 (GraphPad Software, San Diego, CA, USA) and SPSS Statistics 29 Inc. (IBM; Chicago, IL, USA). Changes in eGFR and blood cell counts between baseline and during treatment were analyzed using paired t-test. Univariate Cox regression analysis was employed to assess the predictive potential for earlier and more frequent PR using continuous values. To determine optimal cut-off values for predicting PR, receiver operating characteristics (ROC) curve analysis was performed, with the Youden index applied to identify the most discriminative threshold [18]. These cut-off values were subsequently used in Kaplan-Meier survival analysis (KPM) and group differences were assessed using the log-rank test.

## Results

### Efficacy and safety of [^177^Lu]Lu/[^225^Ac]Ac-PSMA tandem RLT

[^177^Lu]Lu/[^225^Ac]Ac-PSMA tandem RLT led to a median PSA reduction of −12.35% (Interquartile range [IQR], −79,32–64.73%) after two treatment cycles. Similarly, the median reduction in PSMA-TV on follow-up [^18^F]F-PSMA-1007 PET/CT was − 28.14% (IQR, −62.68–99.08%). Based on combined biochemical and imaging response, 9 patients (39%) demonstrated progressive disease (5 both PSA- and PET-based, 2 only PSA-based and 2 only PET-based), 3 (13%) stable disease (2 both PSA- and PET-based and 1 only PSA-based) and 11 (48%) PR (6 both PSA- and PET-based, 1 only PET-based and 4 only PSA-based).

### Safety and tolerability of [^177^Lu]Lu/[^225^Ac]Ac-PSMA tandem RLT

**Fig. 1:**
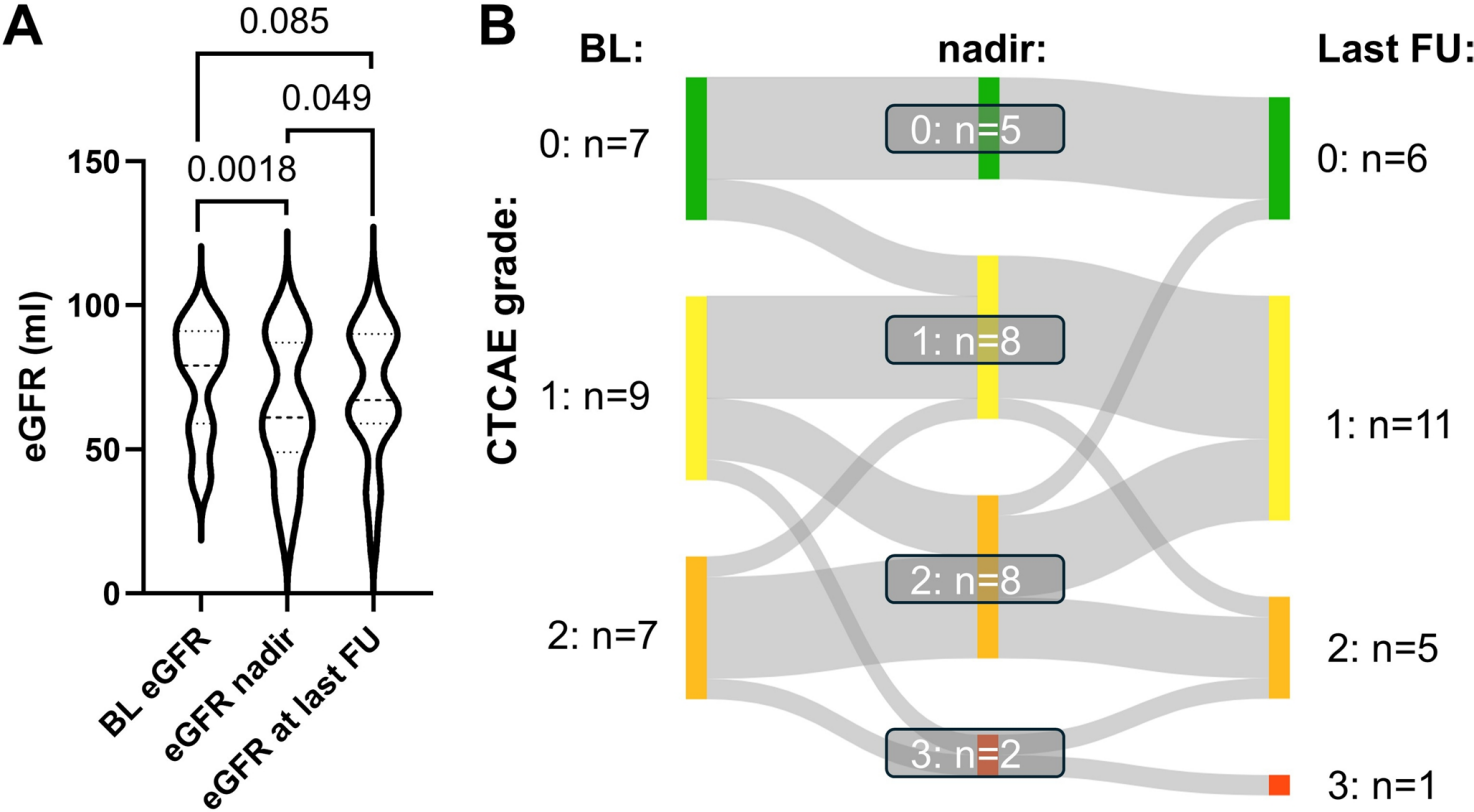
Chronic Kidney Disease (CKD) under Lutetium-177/Actinium-225 prostate-specific membrane antigen tandem radioligand therapy ([^177^Lu]Lu/[^225^Ac]Ac-PSMA tandem RLT).(**A**) Violin plot illustrating the baseline estimated glomerular filtration rate (eGFR), its lowest value (nadir) during tandem-RLT and its value at last available follow-up after treatment termination. (**B**) Sankey diagram showing the distribution of CKD grades, classified following common terminology criteria of adverse events (CTCAE), before, during and after tandem RLT. We observed a statistically significant (*P*=0.0018) decline in eGFR, though with limited clinical relevance. In line, grade 3 CKD occurred infrequently (n=3) and was confined to patients with pre-existing renal functional impairment. eGFR partially recovered after treatment termination (*P*=0.049 relative to nadir)

**Fig. 2:**
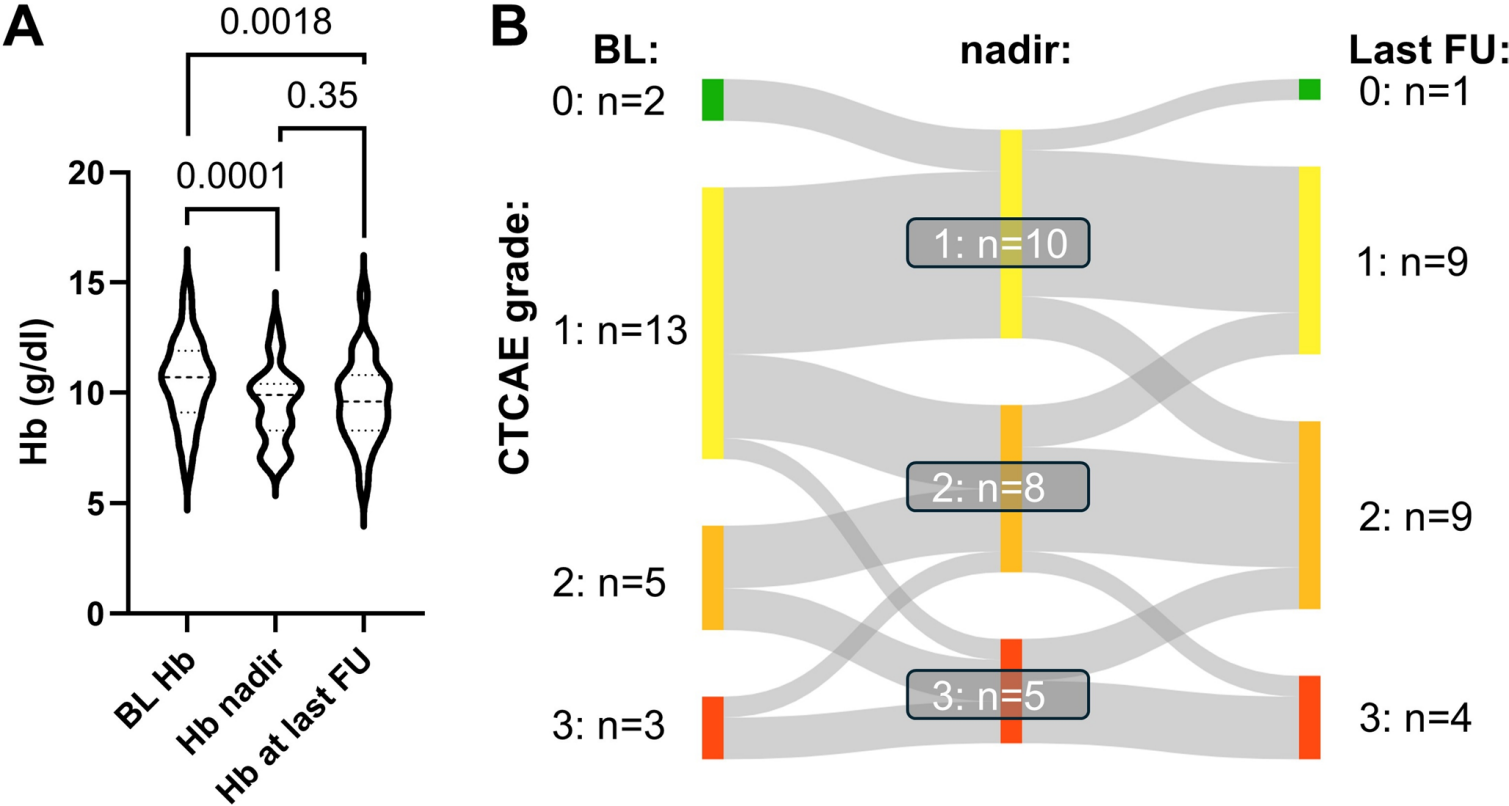
Anemia under Lutetium-177/Actinium-225 prostate-specific membrane antigen tandem-radioligand therapy ([^177^Lu]Lu/[^225^Ac]Ac-PSMA tandem RLT).(**A**) Violin plot illustrating the baseline hemoglobin (Hb), its lowest value (nadir) during tandem-RLT and its value at last available follow-up after treatment termination. (**B**) Sankey diagram showing the distribution of Anemia grades, classified following common terminology criteria of adverse events (CTCAE), before, during and after tandem RLT. We observed a statistically significant (*P*=0.0001) decline in Hb, though with limited clinical relevance. In line, grade 3 Anemia occurred infrequently (n=5) and was confined to patients with pre-existing anemia. Hb remained stable after treatment termination (*P*=0.35 relative to nadir)

**Fig. 3:**
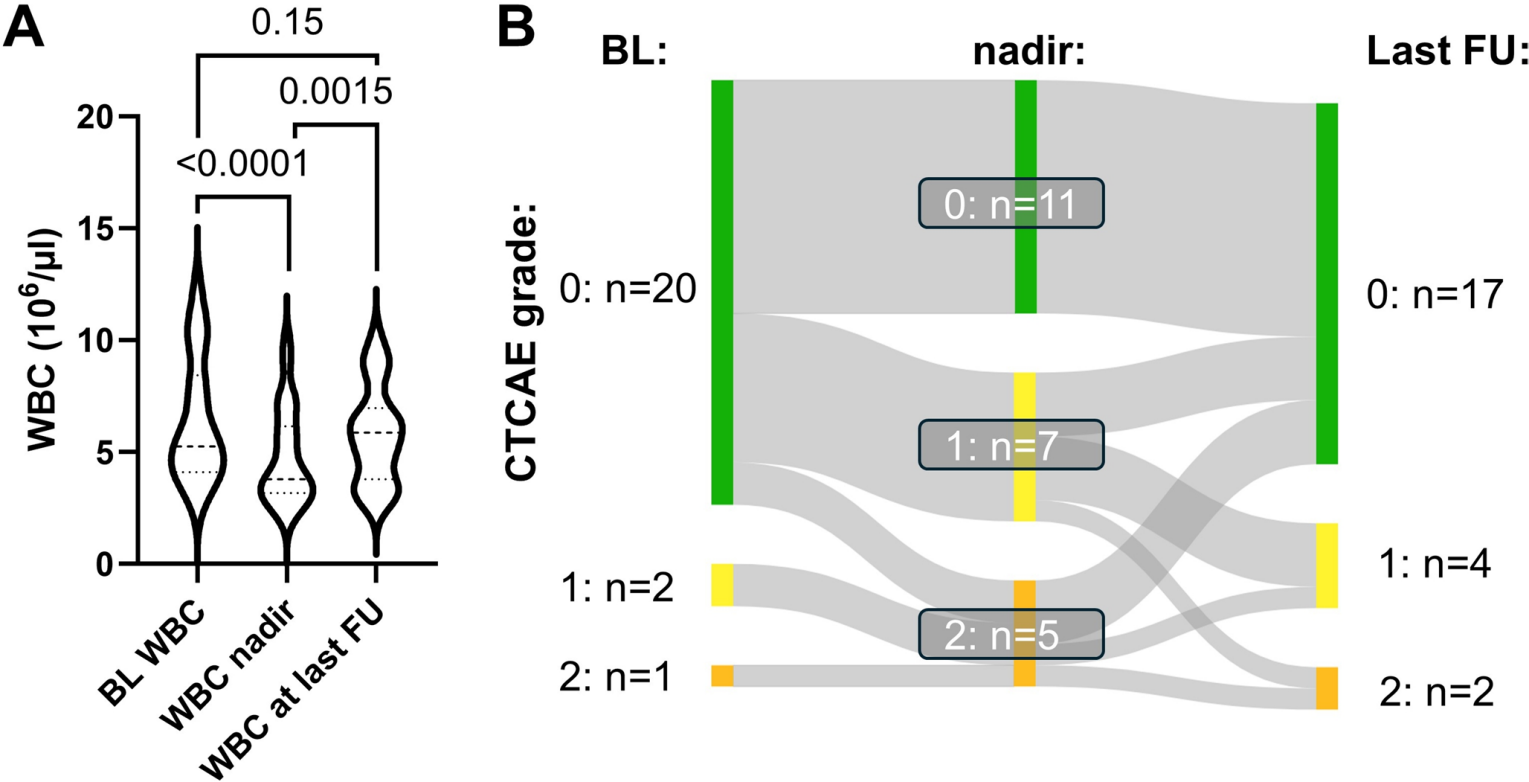
Leukocytopenia under Lutetium-177/Actinium-225 prostate-specific membrane antigen tandem-radioligand therapy ([^177^Lu]Lu/[^225^Ac]Ac-PSMA tandem-RLT).(**A**) Violin plot illustrating the baseline white blood cell count (WBC), its lowest value (nadir) during tandem-RLT and its value at last available follow-up after treatment termination. (**B**) Sankey diagram showing the distribution of leukocytopenia grades, classified following common terminology criteria of adverse events (CTCAE), before, during and after tandem RLT. We observed a statistically significant (*P*<0.0001) decline in WBC, though with limited clinical relevance. There was no leukocytopenia grade 3. WBC recovered after treatment termination (*P*=0.0015 relative to nadir)

**Fig. 4:**
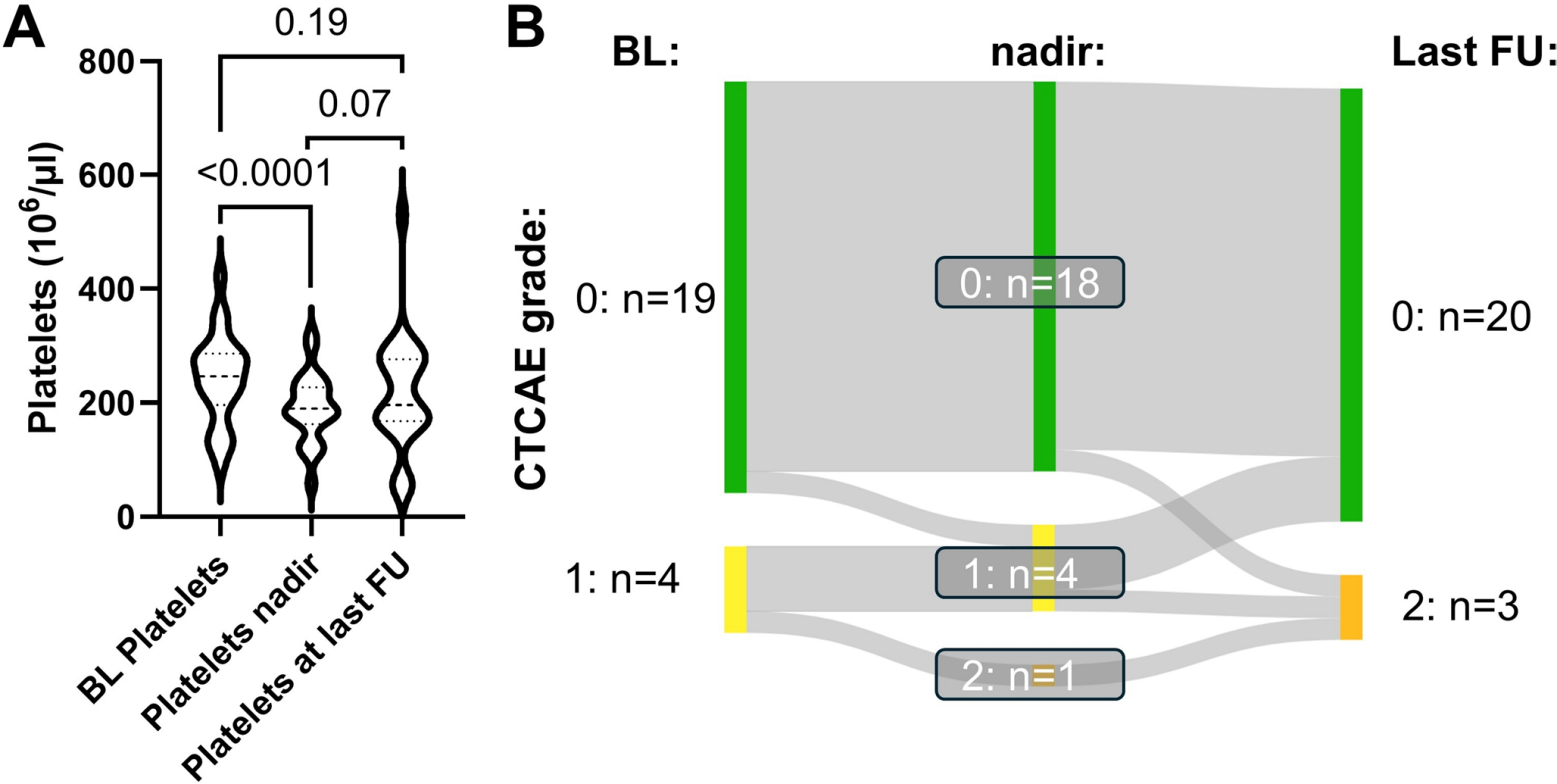
Thrombocytopenia under Lutetium-177/Actinium-225 prostate-specific membrane antigen tandem-radioligand therapy ([^177^Lu]Lu/[^225^Ac]Ac-PSMA tandem RLT).(**A**) Violin plot illustrating the baseline platelets count, its lowest value (nadir) during tandem-RLT and its value at last available follow-up after treatment termination. (**B**) Sankey diagram showing the distribution of thrombocytopenia grades, classified following common terminology criteria of adverse events (CTCAE), before, during and after tandem RLT. We observed a statistically significant (*P*<0.0001) decline in platelets, though with limited clinical relevance. There was no thrombocytopenia grade 3. Platelets remained stable after treatment termination (*P*=0.07 relative to nadir)

[^177^Lu]Lu/[^225^Ac]Ac-PSMA tandem RLT was generally well tolerated. However, we observed significant decreases in eGFR (from 75 ± 20 to 64 ± 24 ml/min/1.73m^2^; *P* = 0.0018), Hb (from 10.6 ± 1.9 to 9.4 ± 1.7 g/dl; *P* = 0.0001), WBC (from 6.4 ± 2.8 to 4.8 ± 2.2 × 10^6^/µl; *P* < 0.0001) and platelets (from 244 ± 80 to 191 ± 60 × 10^6^/µl; *P* < 0.0001) during tandem RLT (Figs. 1, 2, 3 and 4). At last available follow-up after termination of [^177^Lu]Lu/[^225^Ac]Ac-PSMA tandem RLT renal function partially recovered compared to nadir under treatment with an eGFR of 70 ± 23 ml/min/1.73m^2^ (*P* = 0.049; Fig. 1). In addition, WBC also increased after completing RLT (WBC, 5.8 ± 2.2 × 10^6^/µl; *P* = 0.0015; Fig. 3). Hb (9.7 ± 1.9 g/dl; *P* = 0.35; Fig. 2) and platelets (218 ± 103 × 10^6^/µl; *P* = 0.07; Fig. 4) remained stable.

Clinically relevant adverse events of CTCAE grade 3 during RLT included two patients (9%) with CKD and five with anemia (22%), of which all already suffered from renal or bone marrow functional impairment before initiation of tandem-RLT (Figs. 1 and 2). No grade 3 leukocytopenia or thrombocytopenia occurred (Figs. 3 and 4). In addition, we observed no grade 4 or 5 events. Regarding other clinically relevant symptoms during RLT (Table 2), xerostomia was reported in 18 patients, pain in 15 patients and fatigue in 11 patients. Loss of appetite and nausea were less common (each six cases). Of note, these symptoms, especially regarding fatigue and pain, were often already reported before treatment initiation.

**Table 2:** Number of symptoms before and during [^177^Lu]Lu/[^225^Ac]Ac-PSMA tandem RLT

Symptoms	Before treatment	During treatment
Xerostomia	4	18
Loss of appetite	2	6
Nausea	1	6
Fatigue	9	11
Pain	11	15

### PSMA Expression on pretherapeutic PET is predictive for early treatment response

In Cox regression analysis (Table 3), a higher SUV_mean_ was significantly associated with earlier and more frequent partial response, with a hazard ratio (HR) of 1.34 (95% CI, 1.01–1.77; *P* = 0.042). PSMA-TV (*P* = 0.036) and TL-PSMA (*P* = 0.041) also emerged as significant predictors, while SUV_max_ failed to reach significance (*P* = 0.057). Clinical parameters, however, failed to reach significance.

**Table 3:** Cox regression analysis for time to partial response

Variable	Hazard Ratio	95% CI	*P*-value
Clinical parameters at baseline			
Hb (g/dl)	1.068	0.77–1.48	0.69
WBC (x10^6^/µl)	1.001	0.79–1.27	0.99
Platelets (x10^6^/µl)	1	0.99–1.01	0.96
eGFR (ml/min/1.73m^2^)	1.01	0.98–1.04	0.64
AST (U/l)	0.96	0.91–1.03	0.24
ALT (U/I)	0.97	0.91–1.03	0.3
LDH (U/I)	1	1–1	0.53
AP (U/I)	1	0.99–1	0.439
PSA (µg/l)	1	1–1	0.45
PSA doubling time	1.092	0.85–1.41	0.5
Number of previous cycles [^177^Lu]Lu-PSMA RLT	0.92	0.7–1.21	0.56
***PET-derived parameters at baseline***			
SUV_max_	1.051	1–1.11	0.057
SUV_mean_	1.34	1.01–1.77	***0.042***
PSMA-TV (ml)	1.001	1–1.002	***0.036***
TL-PSMA (ml)	1	1–1	***0.041***

Significant parameters are marked in bold and italic. *Hb* hemoglobin, *WBC* white blood cell count, *eGFR* estimated glomerular filtration rate, *AST* aspartate transaminase, *ALT* alanine transaminase, *AP* alkaline phosphatase *LDH* lactate dehydrogenase, *PSA* prostate-specific antigen *PET* positron-emission tomography, *SUV* standardized uptake value, prostate-specific membrane antigen, *TV* tumor volume, *TL* total lesion PSMA

**Fig. 5:**
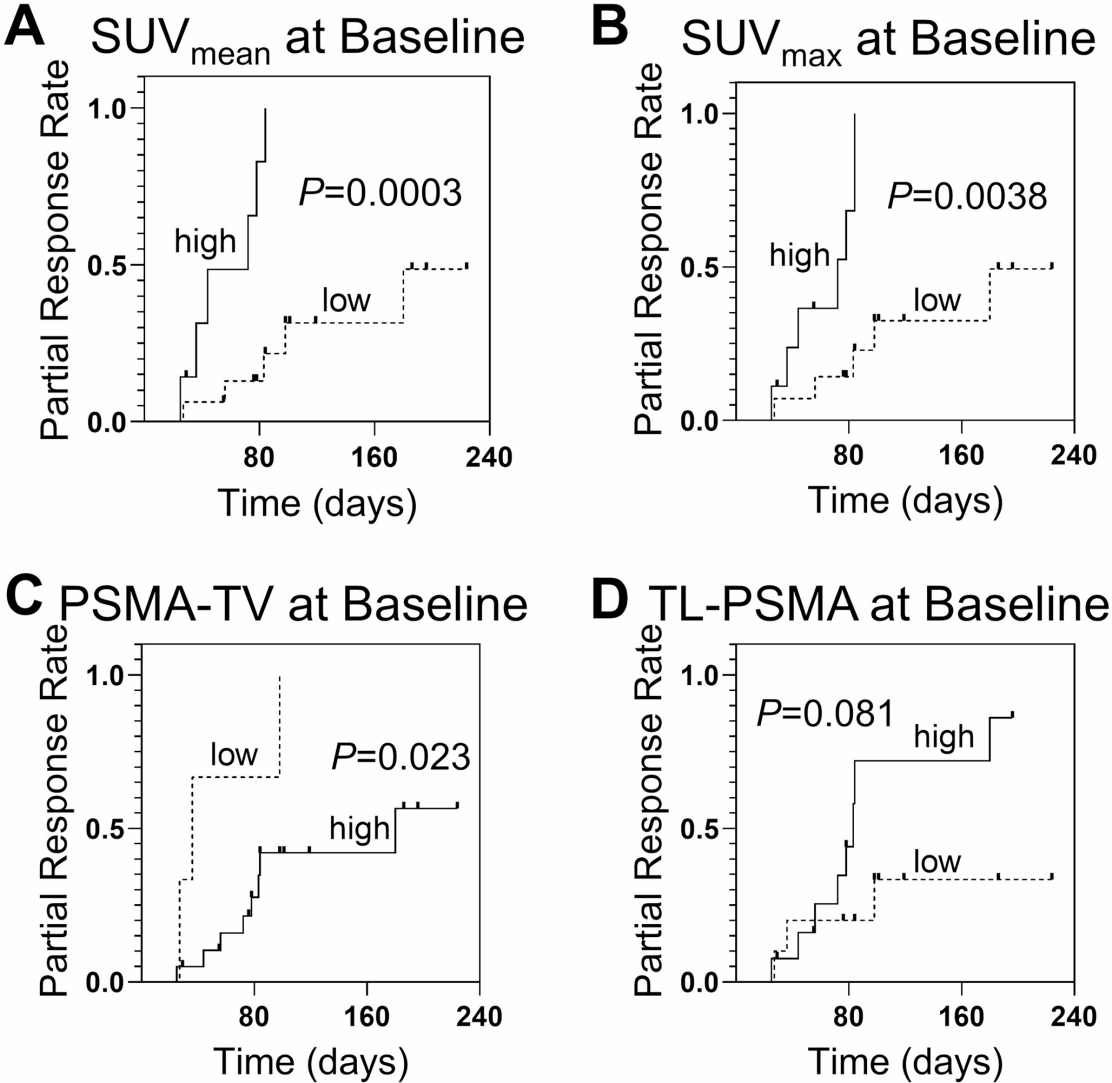
Kaplan-Meier Analysis for Partial Response**. **Higher SUV_mean_ (*P*=0.0003; **A**), higher SUV_max_ (*P*=0.0038;**B**) and lower PSMA-TV (*P*=0.023; **C**) were significantly associated with earlier and more frequent partial response. TL-PSMA (*P*=0.081; **D**) did not reach significance

Using ROC-derived cut-off values (Table 4), 86% (6 out of 7) of patients with high SUV_mean_ reached PR compared to 31% (5 out of 16) in patients with low SUV_mean_. In line with those findings, SUV_mean_ seperated between patients with earlier and more frequent response versus those with delayed or no response (*P =* 0.0003; Fig. 5A) in Kaplan-Meier analysis. In addition, high SUV_max_ (*P* = 0.0038; Fig. 5B) and low PSMA-TV (*P* = 0.023; Fig. 5C) were also linked to favorable outcome. TL-PSMA also tended to reach significance (*P* = 0.081; Fig. 5D).

**Table 4:** Receiver-Operating characteristics

Variable	AUC	*P*-value	Cut-off
SUV_max_	0.583	0.498	15.66
SUV_mean_	0.629	0.295	8.54
PSMA-TV (ml)	0.5	1	114
TL-PSMA (ml)	0.568	0.58	2796

*SUV* standardized uptake value, prostate-specific membrane antigen, *TV* tumor volume, *TL* total lesion PSMA

**Fig. 6:**
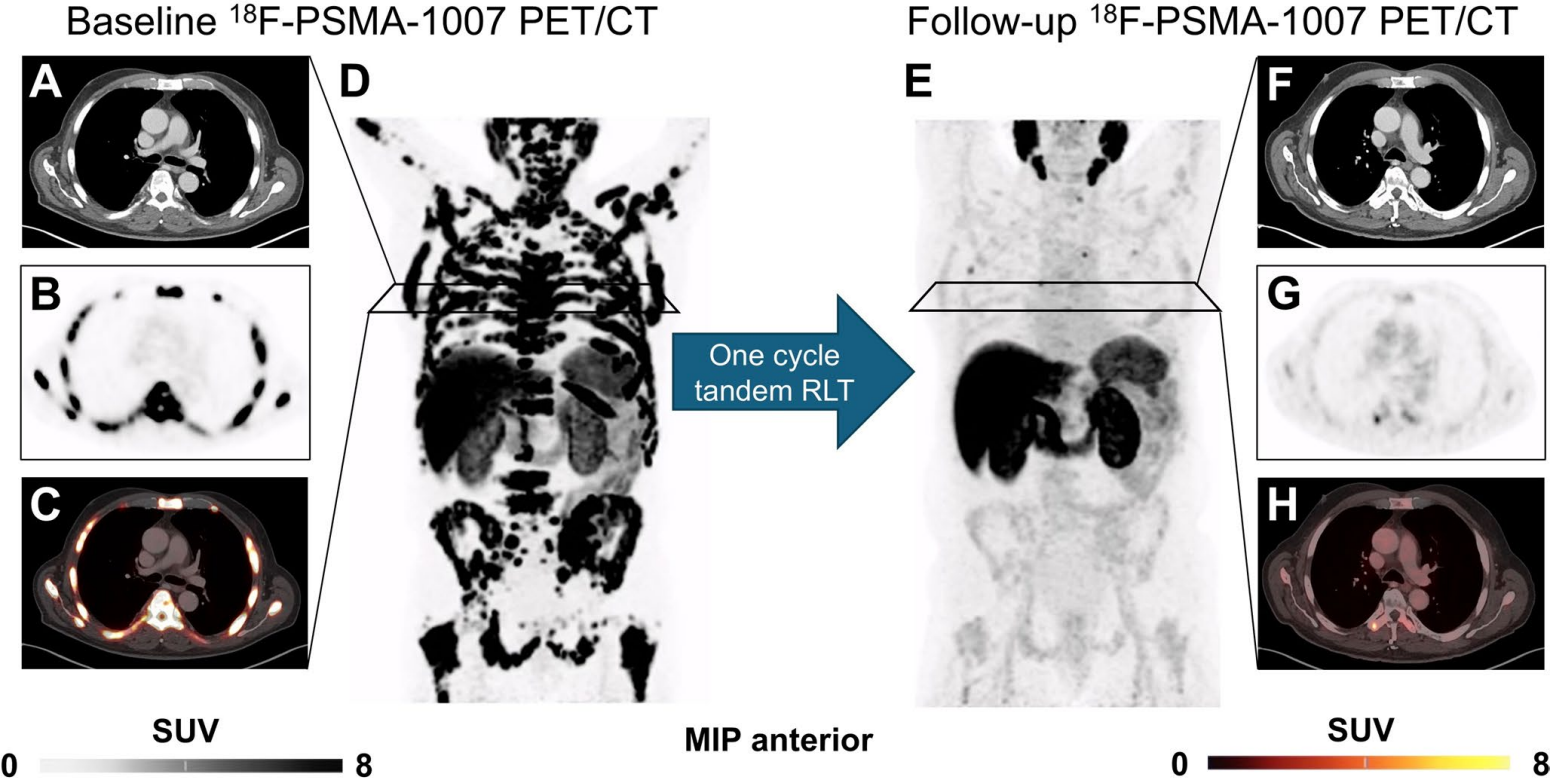
Representative case response to Lutetium-177/Actinium-225 prostate-specific membrane antigen tandem-radioligand therapy ([^177^Lu]Lu/[^225^Ac]Ac-PSMA tandem RLT).Baseline ^18^F-PSMA-1007 PET/CT with maximum intensity projection (MIP, **D**), computed tomography (CT, **A**), positron emission tomography (PET, **B**) and fused PET/CT (**C**). This patient exhibited high PSMA-expression on pretherapeutic PET, notably in the thoracic spine and rips. The Standardized uptake value (SUV_mean_) was above the receiver-operating characteristics derived threshold of 8.54. After only one cycle of tandem RLT prostate specific antigen (PSA) declined from 674 μg/l to 15.6 μg/l (-97.69 %). Follow-up ^18^F-PSMA-1007 PET/CT with MIP (**D**), CT (**F**), PET (**G**) and fused PET/CT (**H**). In line with biochemical response, there was also an imaging-based partial response with PSMA-tumor volume decreasing from 2061.17 ml to 3.36 ml (-99.84%) with absence of new lesions. Despite the extensive tumor burden, the patient did not experience any grade 3 adverse events during treatment

Figure 6 displays a representative case example of a patient demonstrating high PSMA-expression on pretherapeutic [^18^F]F-PSMA-1007 PET/CT (SUV_mean_ above the ROC-derived cut-off value). This patient achieved both biochemical and PET-based response after only one cycle of [^177^Lu]Lu/[^225^Ac]Ac-PSMA tandem RLT, underscoring the predictive value of PET-based PSMA-expression.

## Discussion

The herein presented results underscore the safety and efficacy of [^177^Lu]Lu/[^225^Ac]Ac-PSMA tandem RLT in patients with advanced mCPRC. PR was observed in 11 out of 23 patients (48%) of patients following two treatment cycles. Simultaneously, tandem RLT was generally tolerated well, with only 2 cases (9% of treated patients) of grade 3 CKD and 5 cases (22% of treated patients) of anemia, all occurring in subject with pre-existing renal or bone marrow functional impairment. Moreover, we demonstrate that the PET-based PSMA expression serves as a predictive biomarker for treatment response, supporting its use for individualized patient selection to enhance clinical utility of tandem RLT.

In recent years, targeted alpha therapy has witnessed an expanded use in mCRPC patients [5, 19]. In this regard, α-emitting radionuclides such as Actinium-225 offer significant therapeutic potential due to the higher LET, which induces more effective and complex cellular damage [6]. Consequently, [^225^Ac]Ac-PSMA RLT has shown promising efficacy, even in patients who have progressed following [^177^Lu]Lu-PSMA RLT [5, 20]. However, the intensified radiation from Actinium-225 comes at the cost of increased toxicity, including a higher incidence of CKD and hematological adverse events [5]. Moreover, the high LET is associated with a limited tissue penetration range, which may reduce effectiveness in cases of bulky disease [6].

To address these limitations, tandem RLT combining Lutetium-177 and Actinium-225 has been proposed to leverage their complementary radiobiological properties [7, 8]. Two prior trials investigating single cycle tandem-RLT in mCRPC reported on response rates of 29% and 50%, respectively, which is consistent to the 48% (11 out of 23 patients) in our study [7, 9]. Notably, this is also comparable to the outcome reported for [^225^Ac]Ac-PSMA RLT monotherapy in similar patient populations [21].

Beyond efficacy, our findings also highlight the favorable safety profile of tandem RLT. Of note, while earlier studies were limited to a single cycle of tandem RLT [9], patients in our cohort received up to 8 cycles. To our knowledge, this is the first time that sustained safety of tandem RLT across multiple treatment cycles has been observed. Investigating the safety profile of repeated cycles of [^225^Ac]Ac-PSMA RLT monotherapy, *Feuerecker* et al. report on anemia grade 3/4 in 35% which is slightly higher than the 22% (5 out of 23 patients) in our cohort under tandem RLT [22]. Moreover, they reported on leukocytopenia in 27% and thrombocytopenia in 19%, while there were no such cases in our study [22]. In summary, our findings suggest that our dual radiotherapeutic approach may offer a favorable safety profile, while preserving the potent therapeutic efficacy associated with targeted alpha and beta therapy. Of note, eGFR and platelets even recovered after treatment termination in our cohort, with Hb and platelets at least remaining stable. Nonetheless, to definitely establish the presence of synergistic benefits, future studies, ideally prospective, randomized trials, are needed to directly compare the safety and efficacy of tandem RLT against mono(-beta-)therapies.

Notably, the recently published *VIOLET* phase 1/2 trial by *Buteau* et al. reported on an impressive response rate of 70% under Terbium-161 ([^161^Tb]Tb)-PSMA RLT. Of note, by emitting both β^−^-radiation and high LET short-range Auger electrons, [^161^Tb]Tb-PSMA-RLT may offer similar synergistic therapeutic effects to that seen with [^177^Lu]Lu/[^225^Ac]Ac-PSMA tandem RLT, without the need to combine two radionuclides [23]. Another promising strategy to harness synergistic effects of α- and β^−^-radiation is the use of plumb-212 ([^212^Pb]Pb)-PSMA RLT [24, 25]. As plumb-212 and its decay products emit α-, β^−^- and γ-radiation, this approach enables both effective irradiation of tumor lesions and posttherapeutic imaging via single-photon emission tomography (SPECT) [24, 26]. Following encouraging results in a preclinical mouse model [26], *Berner* et al. recently reported their clinical experience treating three patients with [^212^Pb]Pb-PSMA RLT, demonstrating high tumor uptake [27]. Future studies should aim to compare the efficacy of [^212^Pb]Pb-PSMA RLT with other targeted alpha therapies and tandem approaches.

In our study, we demonstrate for the first time the predictive value of PET-based PSMA expression for early response to tandem-RLT. Specifically, patients with high SUV_mean_ achieved PR in 86% of cases (6 out of 7), compared to only 31% among patients (5 out of 16) with low SUV_mean_. Furthermore, SUV_mean_ was significantly associated with both earlier and more frequent response, as shown by Cox regression analysis (*P* = 0.042) and Kaplan-Meier analysis (*P* = 0.0003). By contrast, traditional biomarkers such as PSA or liver enzymes failed to predict treatment response, which highlights the added value of molecular imaging over routine clinical parameters in patient selection for tandem RLT.

While numerous trials have explored predictors of response to [^177^Lu]Lu-PSMA RLT monotherapy [10], to our knowledge, this is the first study specifically investigating predictors in the context of tandem RLT. Our findings align with earlier reports linking PET-based PSMA expression to treatment response under [^177^Lu]Lu-PSMA RLT monotherapy [11, 28]. However, it can be hypothesizes that factors beyond PSMA-expression, such as tumor radiosensitivity measured on liquid biopsies, may also significantly influence outcomes, particularly in patients exhibiting progressive disease under [^177^Lu]Lu-PSMA RLT monotherapy [29].

This study has several limitations. First of all, the number of enrolled patients was relatively small, and our findings require validation in substantially larger cohorts. Moreover, the retrospective monocentric study design introduces potential biases, including patient selection. Nonetheless, we herein focused on end-stage patients with progressive disease under numerous pretreatments including Lutetium-177 monotherapy. Additionally, the predictive value of PET-based PSMA expression demonstrated in this study was solely based on [^18^F]F-PSMA-1007 PET/CT. Further validation using other PSMA-targeted PET-tracers e.g., [^18^F]F-DCFPyL or Gallium-68([^68^Ga])-PSMA-11 is warranted to confirm the generalizability of these findings.

Finally, PR was chosen as the primary endpoint in this analysis. Unfortunately, overall survival could not be assessed due to frequent loss to follow-up after treatment completion. Nonetheless prior studies have shown that PR correlates with improved overall survival [30, 31], supporting its validation for both early and long-term treatment outcomes under PSMA-targeted RLT.

## Conclusions

The results presented in this study highlight the substantial therapeutic potential of [^177^Lu]Lu/[^225^Ac]Ac-PSMA tandem RLT in patients with advanced mCRPC. The combined use of the α-emitting Actinium-225 and the β^−^-emitting Lutetium-177 appears to offer a synergistic therapeutic effect leading to high efficacy and safety of tandem RLT. In addition, this study emphasizes the predictive value of PSMA expression assessed through pretherapeutic [^18^F]F-PSMA-1007 PET/CT. Specifically, higher SUV_mean_ on baseline PET was significantly associated with earlier and more frequent treatment response. In summary, tandem RLT presents a promising therapeutic strategy in an end-stage setting and integrating imaging-based biomarkers may further refine its clinical utility.

## Data Availability

The herein presented data is available in case of a reasonable request from the corresponding author.
